# The Involvement of Neutrophils in the Pathophysiology and Treatment of Osteoarthritis

**DOI:** 10.3390/biomedicines10071604

**Published:** 2022-07-06

**Authors:** Shelby Chaney, Rosemary Vergara, Zeena Qiryaqoz, Kelsey Suggs, Adil Akkouch

**Affiliations:** 1 Western Michigan University Homer Stryker M.D. School of Medicine, Kalamazoo, MI 49008, USA; shelby.chaney@med.wmich.edu (S.C.); rosemary.vergara@med.wmich.edu (R.V.); zeena.qiryaqoz@med.wmich.edu (Z.Q.); kelsey.suggs@med.wmich.edu (K.S.); 2 Department of Orthopaedic Surgery, Western Michigan University Homer Stryker M.D. School of Medicine, Kalamazoo, MI 49008, USA; 3 Medical Engineering Program, Western Michigan University Homer Stryker M.D. School of Medicine, Kalamazoo, MI 49008, USA

**Keywords:** neutrophils, osteoarthritis, neutrophil elastase, inflammation, cartilage degradation, bone remodeling, microRNAs, exosomes, treatment

## Abstract

Osteoarthritis (OA) is a chronic disability that significantly impairs quality of life. OA is one of the most prevalent joint pathologies in the world, characterized by joint pain and stiffness due to the degeneration of articular cartilage and the remodeling of subchondral bone. OA pathogenesis is unique in that it involves simultaneous reparative and degradative mechanisms. Low-grade inflammation as opposed to high-grade allows for this coexistence. Previously, macrophages and T cells have been identified as playing major roles in the inflammation and destruction of OA joints, but recent studies have demonstrated that neutrophils also contribute to the pathogenesis. Neutrophils are the first immune cells to enter the synovium after joint injury, and neutrophilic activity is indispensably a requisite for the progression of OA. Neutrophils act through multiple mechanisms including tissue degeneration via neutrophil elastase (NE), osteophyte development, and the release of inflammatory cytokines and chemokines. As the actions of neutrophils in OA are discovered, the potential for novel therapeutic targets as well as diagnostic methods are revealed. The use of chondrogenic progenitor cells (CPCs), microRNAs, and exosomes are among the newest therapeutic advances in OA treatment, and this review reveals how they can be used to mitigate destructive neutrophil activity.

## 1. Introduction

OA is a chronic degenerative disease [1,2] and worldwide endemic issue leading to pain, decreased movement, and worsening joint function. While the pathogenesis of this disease is not entirely understood, there are well-established risk factors for developing osteoarthritis such as aging, obesity, female sex, and repetitive movements with excessive loading [3]. Neutrophils contribute via multiple pro-inflammatory and degenerative mechanisms to the progression of OA [4,5,6,7,8] and overall decline of the quality of life of OA patients [9]. This disease affects all synovial joints and is characterized by the progressive destruction of the articular cartilage and secondary episodic synovitis [10]. OA most often affects the interphalangeal joints, hips, spine, knees, and feet [11,12]. It is estimated that worldwide, there are 250 million people who suffer from knee osteoarthritis alone [12]. Predictors of disease include genetics, diet, age, sex, and obesity, with some specific occupations presenting higher rates of OA prevalence than others. In the present review, we assemble recent findings on the involvement of neutrophils in OA pathophysiology, focusing on secreted cytokines, chemokines, metalloproteinases, microRNAs, and exosomes. Understanding the mechanisms of action of neutrophils will contribute to the discovery of new therapies to inhibit the progression of OA and to reestablish joint homeostasis.

## 2. Osteoarthritis Epidemiology

Individuals over 60 years of age are most at risk of developing OA [10,12]. Using 1805 subjects in the Framingham Heart study cohort as an example, adults aged 63–94 years old showed a prevalence of 33% for radiographic knee osteoarthritis and 9.5% for symptomatic knee osteoarthritis [12,13]. In this study, the prevalence of both radiographic and symptomatic knee osteoarthritis increased with age, i.e., from 7.6% in individuals less than 70 years old compared to 15.8% in a population 80 years old or more [13]. In the Chingford Women’s study in the United Kingdom, 13.7% of patients presented with radiographic knee arthritis at a median age of 53 compared to a prevalence of 47.8% at a median age of 68 [10,14]. A cohort of 3000 participants in the United States showed an incidence of radiographic knee OA rising from 26.2% in the 55–64 age group to nearly 50% in the 75 and older age group [10,15]. Overall, age is one of the important risk factors in the development of OA.

Besides age, lifestyle factors can affect the likelihood of developing OA. Obesity is considered a risk factor, with the increased load on the joints potentially leading to undue stress and damage over time [10]. In the Framingham study, women that lost 5 kg had a 50% decrease in risk of symptomatic OA [10,13]. Gender is a potential risk factor, with women being more likely to have OA than men. A study of 3,266,826 participants in Spain showed female risk of acquiring hand OA is greatest after menopause, with 3.5-fold higher rates in women 50–60 years old compared to men of the same age [16]. Several studies have found an association between being female and having OA, but the connection to menopause is unclear [10,16]. In addition to lifestyle and gender, certain occupations with significant mechanical demand have been linked to higher rates of OA.

Osteoarthritis has been a major cause of disability and discharge from the military, affecting tactical athletes for the past decade [17]. A tactical athlete is defined as an individual in a service profession, such as the military or law enforcement, who is required to maintain a standard of physical fitness and performance in order to fulfill their role [17]. While there is less data examining OA specifically in law enforcement personnel and fire fighters, data on military personnel strongly demonstrate an increased risk of developing osteoarthritis of the lower extremity, with a prevalence of 14.21% and 26.91% amongst active-duty service members aged 35–39 and over 40 years old, respectively [17]. Likewise, athletes with heavy physical movement also show an increased risk of OA. A systematic review of 3759 participants found that soccer players (odds ratio OR = 3.5), elite-level long distance runners (OR = 3.3), competitive weightlifters (OR = 6.9), and wrestlers (OR = 3.8) had an increased prevalence of knee OA [18]. With OA being prevalent worldwide among populations and occupations, it is critical to understand the mechanisms underlying this degenerative disease.

## 3. Introduction to Neutrophils and Macrophages Involved in Osteoarthritis

Neutrophils and macrophages are detected within osteoarthritic joints. Macrophages secrete metalloproteinases and inflammatory cytokines while neutrophils secrete degradative proteases including neutrophil elastase (NE), which can cause damage to joint cartilage over time, contributing to the progression of OA [19]. One study showed that NE was not detectable in human OA synovia with no synovitis but was detectable in samples in which “slight” or “moderate” synovitis was present [7]. With regards to abundance in OA joints, macrophages, neutrophils, and T cells can all be found in the synovial fluid and synovial tissue. Macrophages are the most abundant population in synovial tissue, followed by T cells. Neutrophils were the least abundant cell type found within the synovial tissue, found in 35% of patient samples, with a much greater presence in synovial fluid, i.e., a mean of 26% in fluid vs. 8% of total cells in tissue. Another study found that neutrophils made up 8% of the cells in synovial fluid, with no differences in the percentage of cells between men and women [20]. In addition to the presence in synovial fluid, the neutrophil to lymphocyte ratio (NLR) has been associated with OA progression, with a significantly higher NLR seen in patients with severe knee OA compared to those with mild to moderate knee OA [5,21]. Further investigation is needed to elucidate the role that neutrophils, and other immune modulators, play into the progression of OA.

## 4. Normal Bone and Cartilage Formation and Remodeling

During skeletal development, mesenchymal cells are signaled to enter the chondrocyte series under the regulation of the SRY-Box Transcription Factor 9 (Sox9) transcription factor, and then undergo proliferation and hypertrophy during endochondral ossification, a process that is positively regulated by the Runx2 transcription factor. In this series, the proliferative zone is characterized by chondrocytes that produce type VI collagen and become calcified. It is this calcification that provides the foundational structure of endochondral ossification. The matrix will be remodeled and resorbed by matrix metalloprotease (MMP-13) and the activity of osteoclasts and osteoblasts. Alternatively, Runx2 can be inhibited by Sox9, and the immature chondrocyte will become an articular chondrocyte [22]. Notch signaling plays an extensive role in the differentiation, proliferation, and maintenance of different tissues throughout development in a temporal-spatial manner [23,24].

Under normal conditions, bone formation and remodeling are largely controlled by chondrocytes, osteoblasts, and osteoclasts. During intramembranous bone growth, chondrocytes lay down an initial cartilage matrix that is eventually calcified and replaced with mineralized bone. Progenitor cells are induced to differentiate into osteoblasts due to signaling pathways involved osterix (Osx) and Runt-related transcription factor 2 (Runx-2), which is also involved in expression of osteocalcin for the purpose of bone mineralization, osteopontin (OPN) for sealing the zone between osteoclasts and the subosteoclastic compartment, type I collagen which provides compressive strength to bone, and bone sialoprotein for binding osteoblasts to the extracellular matrix [25]. Once mesenchymal cells have differentiated into osteoblasts, these cells will deposit apatite composed of calcium and phosphate as well as type I collagen in an initially immature, haphazard, and unorganized bone matrix that will eventually be remodeled into well-organized Haversian systems in either compact or cancellous bone. Osteoblasts aid in the development and differentiation of osteoclasts, secrete monocyte-colony stimulating factor, express nuclear factor (NF)-kappa B ligand (RANKL) on their surface membrane or as a soluble factor, and inhibit osteoclast activation by producing osteoprotegerin (OPG) which sequesters RANKL, competitively inhibiting its activity [25].

## 5. Osteoarthritis Pathophysiology

**Figure 1:**
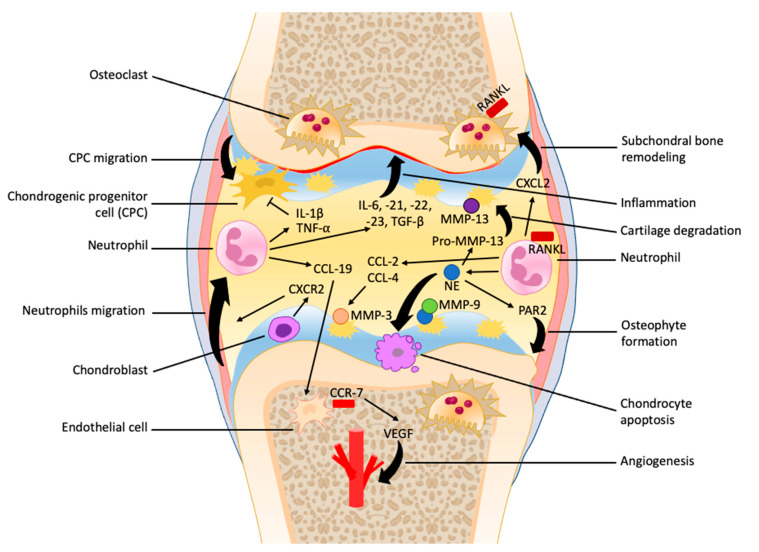
The role of neutrophils in the early as well as late stages of osteoarthritis progression. Neutrophils are recruited at the synovial capsule and contribute to the secretion of many cytokines and chemokines within synovial fluid that promote inflammation and vascular infiltration and inhibit chondrogenic progenitor cell migration. The formation of neutrophil elastase (NE) enhances cartilage degradation, chondrocytes apoptosis, unbalanced subchondral bone remodeling, and osteophyte formation.

The initial stages of OA are characterized by increased compensatory mechanisms, such as chondrocyte hypertrophy and proliferation, increased matrix synthesis, and subchondral bone remodeling [26,27]. As the joint undergoes repetitive insult and the rate of damage surpasses the rate of repair, the cartilage undergoes osteoarthritic change involving decreased chondrocyte proliferation, loss of cartilage, sclerosis of bone, and, in severe cases, osteophyte formation and synovitis [25,26]. The pathological process of OA is heavily influenced by the chronic low-grade inflammation present in the joint, and the involvement of neutrophils is still being researched (Figure 1).

### 5.1. Cartilage Degradation

The compensatory actions of chondrocyte hypertrophy and ECM synthesis occur during the natural process of endochondral ossification and are abnormally activated during the progression of OA. This change in the overlying articular cartilage has been associated with subchondral bone remodeling; however, it is unclear if the changes in bone occur prior to cartilage changes or a result of it [26,28]. Under osteoarthritic conditions, two subpopulations of osteoblasts have been described, i.e., low and high osteoarthritic osteoblasts, which are characterized by low or high secretion of prostaglandin E2 (PGE2) and IL-6. The amount of PGE2 and IL-6 secreted is positively correlated with osteoprotegerin (OPG) expression and negatively correlated with RANKL expression. The ultimate effect is that low osteoblasts allow higher rates of bone resorption compared to high osteoblasts [25]. Inflammatory mediators released from immune cells, such as neutrophils that have extravasated to the site of injury, play a role in these morphologic changes as well. Neutrophils contribute to this bone resorption by activating osteoclasts through increased RANKL expression on neutrophils and induction of RANKL secretion from osteoclast precursors. Neutrophilic RANKL expression is induced by Toll-like receptor 4 (TLR4) activation, while osteoclast precursor RANKL secretion is induced by the neutrophil chemoattractant chemokine (C-X-C motif) ligands 2 (CXCL2) [6].

In late-stage OA, unrestricted chondrocyte differentiation, proliferation, and hypertrophy results in the calcification and sclerosis of subchondral bone accompanied by articular surface fibrillation [26,28]. As OA progresses, hypertrophic chondrocytes undergo apoptosis and halt proliferation. The resultant lacunar emptying after apoptosis leads to loss of articular cartilage and eventually osteophytes [26]. The proposed mediator causing irreversible cartilage degradation in OA is overactivated MMP-13 [25]. Neutrophils play a role in activating latent pro-MMP-13 through the release of NE. Even at low concentrations, NE has been shown to degrade cartilage collagen quickly in vitro [7]. An in vivo murine model demonstrated that it only took 4 h for NE incubated with cartilage to compromise its structure and cause significant pain development [29]. NE also inhibits chondrocyte proliferation and promotes apoptosis. This is shown by reduced survival of chondrocytes exposed to NE in a dose-dependent manner [1]. NE is hypothesized to induce apoptosis by means of caspase 3 activation, inducing DNA degeneration, increasing free calcium levels, disrupting mitochondrial membrane potential, and increasing intracellular reactive oxygen species (ROS) production [1]. As receptive insult to the joint and the continued weakening of cartilage continues, fibrillation and micro-fractures occur and cause damage of the underlying subchondral bone. This leads to inflammation, bone remodeling, unresolved edema, and eventually, osteosclerosis. There is no proven mechanism of inflammation inducing osteosclerosis. B cells and macrophages have shown to be significantly increased in sclerotic bone. However, when studies used macrophage-depleted murine models in post-trauma OA, the injured joint was not recovered. In fact, this led to higher levels of systemic inflammation and increased levels of T cells and neutrophils extravasated into the joint [30]. This suggests that macrophages have a somewhat protective, anti-inflammatory phenotype to inhibit the injury inflicted by neutrophilic activity. Sclerotic bone ultimately leads to osteophyte formation, as an attempt to distribute the burden of stress on the joint by increasing the surface area [31]. NE was implicated in the development of osteophytes through the activation of proteinase-activated receptor 2 (PAR2). A murine study showed that PAR2 deficiency significantly reduces the presence of osteophytes, further exposing that OA pathology requires neutrophilic activity [5]. However, there have been contradicting results from clinical OA studies that used colchicine to inhibit neutrophil activity [5]. One randomized control trial of OA patients found no significant difference in inflammatory markers between the colchicine and placebo treated groups [32]. This suggests that neutrophils are necessary for recovery, but in OA pathology, they exhibit erroneous activity. This erroneous activity of neutrophils may be mediated by increased mechanosensory signals within the joint, such as shear stress, that lead to activation via mechanotransduction of neutrophils and subsequent degeneration of cartilage [33].

### 5.2. Synovial Tissue

OA has been shown to affect the synovial tissue of joints causing inflammatory synovitis. Synovitis does not always occur during OA, but when it is present, it is correlated with more extreme pain, joint dysfunction, and more rapid cartilage deterioration. Synovitis severity increases as OA progresses, characterized by cartilage lesions and radiographic alterations [30]. Inflammation in the OA synovium has previously been linked to macrophages. However, recent studies have shown that neutrophils play a significant role in the propagation of synovitis [5].

Immune cells, fibroblasts, and well vascularized connective tissue contribute functional units to the synovial fluid. These cellular components undergo histological changes during synovitis such as synovial lining hyperplasia, mononuclear cell invasion, neoangiogenesis and capsular fibrosis. After cellular stress and extracellular matrix (ECM) defacement, the innate immune system is activated through toll-like receptors (TLRs) which then activates the transcription factor nuclear-factor kappa B (NF-ΚB). NF-ΚB plays a significant role in producing proinflammatory cytokines that recruit macrophages, granulocytes, and lymphocytes. These immune cells induce the catabolism of chondrocytes [30]. Neutrophils are among the first immune cells to infiltrate into the synovium during OA [28]. However, macrophages were found to be the most abundant immune cells in synovial fluid and the synovium [5]. Neutrophils are less abundant but may have the most substantial cytotoxicity among all the immune cells present in OA. For example, degradative elastase is significantly correlated with OA severity and neutrophils were shown to be the predominant source [5]. Furthermore, neutrophilic elastase may allow neutrophils as well as other immune cell types to transmigrate across the ECM and augment the extent of inflammation. Neutrophils also promote cartilage degradation as a consequence of forming a complex with MMP-9 and neutrophil gelatinase-associated lipocalin in the synovial fluid [5].

Neutrophils contribute to the many cytokines and chemokines released by immune cells within synovial fluid that promote OA progression. Neutrophilic cytokines found in the synovial fluid of OA joints include interleukin (IL)-1β, IL-6, IL-21, IL-22, IL-23, tumor necrosis factor (TNF)-α, and transforming growth factor (TGF)-β [5,8]. Increased levels of IL-7 have also been found in synovial tissue, which is known to precipitate the recruitment of neutrophils [31]. The different functions of these cytokines include pro-inflammatory (IL-1β, IL-6, IL-22, and TNF-α) and immunoregulatory (IL-21, IL-23, and TGF-β) [8]. The presence of these 2 opposing classes of cytokines reveals the complex balance of pro- and anti-inflammatory activity that gives rise to the characteristic low-grade inflammation in OA and permits the combination of restorative and degradative processes [4]. Thus, the inhibition of just one cytokine may not be enough to ameliorate the pathology of OA. For example, TNF-α is readily present in the joints of OA patients and incites chondrocyte catabolism in vitro. However, the use of TNF inhibitors does not significantly improve pathology. Nonetheless, some OA patients reported melioration of pain and function [30]. This suggests that utilizing cytokines as therapeutic targets will require a complete understanding of the balance and interactions of all cytokines involved in OA pathogenesis.

Chemokines are involved in the recruitment of immune cells and activation of signaling cascades in the synovium and synovial fluid of patients with OA synovitis. Molnar et al. suggests that the most substantial chemokine families affiliated with OA are C-C motif chemokine ligand (CCL)2, CCL3, CCL4, CCL5, C-X-C motif chemokine ligand (CXCL)8 (IL-8) and CXCL12. Neutrophils are known to produce all of these except CCL5 and CXCL12 [4]. Chemokines like monocyte chemoattractant protein (MCP-1) (CCL2) and macrophage inflammatory protein (MIP-1β) (CCL4) have been linked to joint pain, possibly due to proteoglycan loss in articular cartilage, which is caused by an upregulation of MMP-3 [34]. MCP-1 and MMP-3 are significantly correlated with the presence of neutrophils and macrophages [35]. Scanzello et al. additionally found CCL19, CCL21, C-C motif chemokine receptor (CCR)7, and C-X-C motif chemokine receptor (CXCR)2 involved in OA synovitis [36]. CCL19 and CCR7 are correlated with more severe symptoms and are markers of early synovitis. CCL19 activates CCR7 expression on synovial fibroblasts which then stimulates the release of vascular endothelial growth factor (VEGF) leading to neoangiogenesis in synovial tissue [34]. Neoangiogenesis characterizes tissue healing but may have detrimental effects on normally avascular cartilage [37]. Neutrophils are known to produce CCL19 and VEGF in larger amounts than macrophages or lymphocytes [8]. Neutrophils have been shown to house an intracellular pool of VEGF, with considerable amounts of VEGF detected in supernatant after vesicle release post-stimulation [38]. Plasma concentrations of VEGF in OA patients parallel the synovial fluid levels which suggests it may be a helpful biomarker of severity [35]. VEGF, in fact, has been linked with increasing grade of OA severity, higher rated degree of OA pain, and is associated with increased vascular density and endothelial cell proliferation within OA synovium [39]. Another, inflammatory mechanism that chemokines facilitate is the recruitment of immune cells. CXCL8 and CXCR2 are known neutrophil chemoattractants [36,40]. CXCR2 is expressed on articular chondrocytes; specifically, it enables the chemotaxis of neutrophils when bound by ligands [36]. Neutrophils are the first site of inflammation and therefore have some control over the initial inflammatory response [41]. Considering the substantial impact of cytokines and chemokines on the synovial environment, they may be utilized as therapeutic targets and biomarkers to improve OA treatment and evaluate disease severity [34]. Novel therapeutic methodologies including chondrogenic progenitor cells (CPCs), microRNAs, and exosomes aim to target the pathological processes discussed above and many are related to moderating harmful neutrophilic activity.

### 5.3. Chondrogenic Progenitor Cells

After an injury that results in chondrocyte death, hypocellularity occurs. Hypocellularity may be due to chondrogenic progenitor cells (CPCs) migrating from the surrounding matrix and proliferating. CPCs are multipotent, clonogenic and chemotactic like mesenchymal stem cells (MSCs) but are more limited in their differentiation capacity compared to MSCs. CPCs contain stem cell-associated markers and have the potential to repair injured cartilage and attenuate inflammation [42,43]. Some studies suggest that CPCs play a role in cartilage repair by forming a continuous sheet on the injured surface and increasing proteoglycan 4 (PRG4), which recovers a surface-protective lubricant [42]. CPCs are not resident in cartilage tissue and must migrate to the site of injury. CPC migration is mediated by the chemoattractant high mobility group box I protein (HMGB1). HMGB1 binds receptor for advanced glycation end products (RAGE) and TLR4 which are both present in CPCs as well as OA tissue and chondrocytes [44]. A study showed that IL-1β and TNF-α inhibited the migration of CPCs [45]. These cytokines are known to be upregulated in an OA joint and are known to be released by neutrophils, therefore IL-1β and TNF-α may contribute to the lack of repair [5,46]. CPCs have also been studied to assess their immunomodulatory effects and functional similarity to MSCs. Luca et al. found that CPCs have both pro- and anti-inflammatory effects, but the anti-inflammatory is more pronounced. For example, CPCs produce IL-1RA, which is a direct inhibitor of IL-1β [37]. The immunomodulatory activity of CPCs is still on going, yet the current knowledge offers prospective anti-inflammatory treatment.

However, some studies have shown that macroscopic cartilage lesions do not heal properly even with CPCs present. Additionally, CPCs may contribute to synovitis and cartilage loss via increasing levels of deleterious cytokines, chemokines and MMPs [36]. These contradictory phenotypes of CPCs suggest a mechanism of alternative activation in response to varying environmental conditions. The function of these CPCs may be altered using HMGB1 to stimulate cartilage regeneration [36]. During endochondral ossification, hypertrophic chondrocytes secrete HMGB1 and in the context of OA, this contributes to pathologic calcification [37]. The relation of CPCs to inflammation and specific biological mediators tangentially implicates the inhibition of neutrophils in OA treatment.

### 5.4. MicroRNAs

MicroRNAs (miRNAs) are small non-coding RNAs that are used as biomarkers and post-transcriptional gene expression modulators in many pathological conditions, including osteoarthritis. They function by regulating gene expression post-transcription through base pairing with target miRNAs. The implications of miRNAs as regulators of gene expression and modulating pro-inflammatory neutrophil functions are beginning to emerge. Multiple miRNAs are secreted by neutrophils and can be up or downregulated in OA environments (Table 1). For example, certain miRNAs such as miRNA-141 have been theorized to play an important role in the inhibition of resorption of bone. Along with neutrophil-lymphocyte ratio (NLR), miRNA-141 levels were found to be elevated in patients with osteoarthritis. This finding was proven to have important diagnostic value and paves the way for potential therapeutic targets for OA [39]. Further dive into literature reveals that overexpression of miRNA-451 was associated with decrease in neutrophil chemotaxis and in turn attenuating arthritis severity [40]. It has additionally been found that miRNAs could have an impact in regulating cytokine secretion in neutrophils: miR-146a-5p and miR-155-5p overexpression was found to reduce S100A8/A9-P secretion of proinflammatory cytokines [41]. The creation of a comprehensive, OA-specific miRNA interactome was noted to have significant differential expression between lesioned and preserved cartilage [47]. This technique allowed for identification of certain miRNAs including miR-99a-3p, which is downregulated and targets a plethora of mRNAs (Frizzled Class Receptor 1 (FZD1), Integrin Subunit Beta 5 (ITGB5), and growth differentiation factor 6 (GDF6)), while miR-143-5p is increased and targets mRNAs associated with genes like SMAD3 and dephospho-CoA kinase domain-containing protein (DCAKD) [47]. These findings suggest that miRNAs in OA impact the downstream secretion of inflammatory cytokines and further enhance OA progression.

As alluded to earlier, miRNAs may be manipulated in ways intended to supplement the repair processes of cartilage or hinder the progression of OA due to their diverse roles in regulating posttranscriptional genes and inflammation, autophagy/apoptosis, and chondrogenesis [50]. MiRNAs that are upregulated and contribute to the inflammation of OA include: miR-146a which aggravates pro-inflammatory cytokines, targets calcium/calmodulin-dependent protein kinase II delta (Camk2d) [50,58] and miR-136-5p by inducing expression of inflammatory factors and chemokines via NF-ΚB/A20 signaling [53]. MiRNAs that are downregulated, and normally attenuate inflammation include miR-9 which is a target of IL-6, NF-ΚB1, and MMP-13 [48]. To illustrate miRNA’s involvement in OA inflammation, one in vitro study found that miR-9 reduced LPS-induced inflammation and damage in a murine chondrocyte cell line [49]. These results indicate that altering specific miRNAs can improve inflammation caused by the OA microenvironment.

MiRNAs that are upregulated and involved with cell survival and apoptosis in OA include miR-155 (inhibits autophagy) and miR-30 (inhibits autophagy, induces apoptosis) [42,46]. One miRNA that is downregulated and does the opposite of these is miR-146a, which reduces type II collagen, enhances autophagy, and inhibits the NF-ΚB pathway [42]. For instance, a randomized control trial was conducted to study the effect of Xinfeng capsules (XFC) on the miR-146/NF-ΚB pathway in OA patients. The treatment group saw an improvement in blood stasis and a decrease in pro-inflammatory cytokines. The researchers concluded that XFCs improved OA through the upregulation of miRNA-146 and the NF-ΚB pathways it inhibits [47]. Evidence from this clinical trial suggests that upregulating specific miRNAs could improve an OA cellular environment in vivo.

MiRNAs that are upregulated and interfere with chondrogenesis include miR-29 (represses SRY-box transcription factor 9 (SOX9), targets collagen type 1 alpha 1 (COL1A1)), miR-1271-5 (associated with matrix-degrading components), miR-21 (inhibits growth differentiation factor 5 (GDF5)), and miR-490-5p (increases Runx2, decreases SOX9, influences human adipose derived stem cell (hADSC) osteogenic differentiation) [50,51,52]. MiRNAs that are downregulated and normally improve chondrogenesis include: miR-204 (increases Runx2), miR-222 (regulates MMP-13 and histone deacetylase 4 (HDAC4)), miR-140 (induces SOX9, suppresses hypertrophy by targeting SMAD1), miR-92 (inhibits HDAC), miR-381 (inhibits HDAC), miR-370/373 (inhibit serine hydroxymethyltransferase 2 (SHMT2) and methyl CpG-binding protein 2 (MECP2)) [50,54]. For example, one study used CircSERPINE2 to inhibit miR-1271-5 expression in human chondrocytes resulting in a decrease in the progression of OA [51]. Evidence from this study suggests that miRNAs may be a novel target in OA treatment and a tool for the diagnosis of OA progression.

### 5.5. Exosomes

Exosomes are extracellular microvesicles derived from body fluids or cells which are used as intermediate cell mediators, biomarkers, or therapeutics for various pathologies [51]. Exosomes developed from mesenchymal stem cells, bone cells, or chondrocytes have been identified as important players in bone and cartilage regeneration [59]. Exosomes contain various miRNAs, long non-coding RNAs (lncRNAs), tRNA fragments (tRFs), and other soluble mediators which have been associated with the progression or prevention of OA pathologies [55]. Exosome research in OA is bipolar in that it focuses on either the diagnostic significance of endogenous exosomes or the therapeutic effects of stem-cell derived exosomes on OA [60]. However, in a study by Zhan and colleagues, it was found that neutrophil-derived microvesicles can be internalized by fibroblast-like synoviocytes and down-regulated TNF-α induced expression of IL-5, IL-6, IL-8, MCP-1, IFN-γ and MIP-1β [61].

Endogenous exosomes are currently used as biomarkers for other diseases such as cancer onset and progression, and studies are attempting to apply this technique to OA detection. Endogenous exosomes can be extracted from the synovial fluid, blood or urine and identified by morphological features, size, and surface markers. These surface markers include CD9, CD63, CD81, and heat shock protein 90 (HSP90) [51]. Exosomes that may be used as biomarkers, contain molecules associated with OA progression through pro-inflammatory action, and multiple mechanisms of chondrogenesis inhibition [49]. These molecules include: lncRNA-PCGEM1 (gradually elevated with OA progression), tRF-25, tRF-38, tRF-28 (these tRFs are increased in osteoporosis), and elevated levels of miRNA-181d-3p, 3904-3p, 155-3p, 4532, 185-5p, 7107-5p, 6865-3p, 4459, and 71-7-5p in synovial fluid-derived exosomes from female OA patients [51,52]. For example, exosomes’ diagnostic significance was discovered in end-stage knee OA, when synovial fluid-derived exosomes were found to have a higher level of inflammatory cytokines and chemokines suggesting that these exosomes recruit inflammatory cells and inhibit cartilage proliferation [54]. The use of exosomes as biomarkers for OA is relatively new and more research is necessary to identify subgroups of exosomes [51].

The therapeutic use of MSC-derived exosomes attempts to utilize RNA and cytokine contents to improve the inflammation and cartilage degeneration in OA. MSCs have been used in many recent studies because of their functions in cartilage repair and inflammation attenuation [55]. In analyzing exosomes secreted by human bone marrow stem cells (hBMSCs) differentiated into cartilage, Sun et al. found that miRNAs including miR-1246, miR-1290, miR193a-5p, miR320c, and miR-92a were upregulated. They further found that the exosome derived miR-320c worked to enhance chondrogenesis by upregulating SOX9 and downregulating MMP-13. associated with OA improvement include: miR-100-5p (cartilage homeostasis), miR-135b (chondrocyte proliferation and cartilage repair), miR-92a-3p (cartilage development/homeostasis), miR-95-5p (cartilage development/homeostasis), miR-140-5p (proliferation and migration of chondrocytes), and lncRNA KLF3-AS1 (cartilage repair and chondrocyte proliferation) [55]. To show the use of MSCs and exosomes in cartilage repair, one study preconditioned MSCs with TGF-β1 causing an increase in MSC-derived exosome miR-135b which then improved cartilage proliferation and repair [57]. OA improvement was also shown in a murine model, using MSC-derived exosomes containing miR-92a-3p. The significant results included: slowing of OA progression, increased cartilage proliferation and stability, and inhibition of cartilage degradation [56]. Exosomes are a great prospect for safe, efficacious clinical application since they have the advantages of advanced intracellular communication and no cell structure which decreases the chances of immune rejection [55].

## 6. Current Treatments & Investigations

As osteoarthritis is not currently curable, the goal of all OA treatment is to manage symptoms, slow the progression of disease and improve daily quality of life. Lotz describes three phases of OA; an immediate phase after injury, an acute phase lasting up to two months, and a chronic phase that may persist for years. The immediate phase can be characterized by lesions within the joint, including but not limited to fracture, ruptured ligaments, meniscal injury, or shear or compressive damage affecting the articular surface. The result of this compressive destruction is cartilage dislocation and necrosis and may include hemarthrosis. In response to immediate mechanical damage, neutrophil-derived-enzymes break down lubricin, one of the major joint lubricants in addition to hyaluronic acid, with other inflammatory mediators further suppressing the production of lubricin [62]. Interestingly, hemarthrosis can induce chondrocyte apoptosis, while inflammatory infiltrate that accompanies hemarthrosis produces mediators, including reactive oxygen species, that can directly impair cartilage as well. Chondrocyte apoptosis, which may be focal initially, can spread to otherwise unblemished areas of cartilage, which may further exacerbate the initial injury, and may begin a cascade that contributes to the delayed progression of OA. Intervening in this apoptotic pathway, then, represents a potential therapeutic avenue for delaying, treating, or preventing the development of OA. Caspase inhibitors have been investigated in vitro and have been associated with genes encoding for cartilage/ bone pathologies such as OA [63].

At the time a patient seeks a diagnosis, they are experiencing issues with limited mobility, pain of the joint, and stiffness. It is critical to note that OA is traditionally diagnosed by a physical exam and radiographic imaging to detect bony deformities, with less common usage of magnetic resonance imaging (MRI) and ultrasound [64]. By the time patients experience pain and have eburnation present on imaging, they are commonly diagnosed with mid to late cartilage degradation. This advanced stage of joint issues limits the options available for treatment on a case-by-case basis and has sparked interest in developing criteria to diagnose OA at an earlier stage to improve treatment outcomes [65,66]. Current treatment of OA consists of a mix of weight loss if applicable, physical therapy, pharmacological interventions, and surgical procedures [67]. Lifestyle interventions for the treatment of OA include exercise, weight loss, orthopedic aids, and physical therapy. Orthopedic aids, such as shock-absorbing shoe inserts, can remove some mechanical stress affecting the joint [67]. Physical therapy is currently a primary treatment for patients with OA, especially of the hip and knee [68]. Exercise therapy strengthens the muscles and increases stability around affected joints, with a regimen of at least 12 supervised sessions occurring twice a week to begin seeing clinical improvement [68].

Physiotherapeutic measures include electrotherapy, acupuncture, ultrasound, and the application of heat/cold [67]. While there is limited medical evidence supporting their efficacy, it is important to note that many homeopathic medicines exist for the treatment of OA in the form of ointments, herbal creams, and special diets with high levels of gelatin and amino sugars [67]. The main goal of homeopathic remedies is to decrease pain and inflammation and improve the mobility of the affected joint. While there is limited evidence, some examples of homeopathic remedies that show potential to be further investigated in the future include Avocado soybean unsaponifiables, calcarea carbonica, boswellia serrata, and ginger [69,70,71].

The goal of pharmacological interventions is to improve pain and decrease inflammation of the affected joints. Non-steroidal anti-inflammatory drugs (NSAIDs) are prescribed to alleviate pain of the affected joint, with some relief of inflammation. In addition to NSAIDs, current treatment options include glucocorticoids, opioids, and cytokine inhibitors [67]. Intra-articular glucocorticoid injections are a critical treatment tool to rapidly eliminate a joint effusion but need to be used with caution in diabetic patients who are already hyperglycemic [67]. Slow-acting drugs in osteoarthritis (SADOA) drugs include those that do not inhibit prostaglandin synthesis like NSAIDs and include hyaluronic acid, D-glucosamine sulphate, and diacerein [67]. Anti-cytokine treatments include the use of antibodies against pro-inflammatory cytokines like TNF-α or the use of anti-inflammatory cytokines such as IL-4, IL-10 and TNF-β [62,67].

Cytokine inhibitors have been investigated in osteoarthritis patients. Several studies have investigated the effect of modulating IL-1 activity, a cytokine linked to cartilage degradation and initially promising candidate for application to OA that can be produced by immune cells including neutrophils [72,73]. Preliminary data from clinical trials shows administration of IL-1Rα, a receptor antagonist of IL-1, may provide symptomatic relief of osteoarthritis, result in less osteolysis and bone tunnel enlargement, and slow progression of disease [74,75]. However, there have been multiple studies that also show IL-1 blockade provide little to no alleviating effects of OA symptomology or pain [76,77]. More investigations need to be conducted into the realm of IL-1 blockade and other cytokine therapies to determine the overall applicability of this treatment in the context of OA. Lastly, bone morphogenic proteins (BMPs), specifically BMP-7, may also be beneficial in treatment of OA, and has been shown in various animal models of cartilage defects to lead to regeneration of articular cartilage as well as improve the interaction between new cartilage and the healthy cartilage of the articular surface [62]. When all lifestyle and pharmacologic interventions have been exhausted, surgical intervention may be necessary. Surgical options include an arthroscopic lavage, shaving, debridement, bone microfracturing, and abrasion arthroplasty [67]. Additionally, joint restoration procedures include autologous transplantation with either chondrocytes or osteochondrocytes [67]. A recent study explored whether neutrophils could play a role in increasing complications after osteochondral grafting. Using animal trials, the authors concluded that antimicrobial neutrophil extract may be considered as a tool for regulating the neutrophil response post-transplantation, promising for future investigation [78].

## 7. Future Directions

OA is a prevalent worldwide issue. With increased prevalence of musculoskeletal injuries, an aging population, and higher rates of obesity, it is to be expected that more individuals will be diagnosed with OA in the future. As such, there is a need to understand the inflammatory pathways that underlie this disease in order to monitor OA progression and develop new forms of interventions. The mix of pro-inflammatory and anti-inflammatory cytokines in the synovial fluid of OA patients, as well as the presence of distinct chemokines, reveal a dynamic microenvironment in a balance of restorative and degradative processes.

Neutrophil function is associated with inflammation and disease progression in OA, making investigations in this field a promising area of discovery [5,19,35]. Beyond neutrophils, additional areas of investigation for the treatment of OA include liposomal nanoparticles and mesenchymal stem cell-based therapies [79,80]. Future investigations of OA need to consider the dynamic balance of the OA microenvironment to fully encapsulate the complexity of this disease.

## Figures and Tables

**Table 1:** MicroRNAs differentially expressed in OA resulting from potential impaired expression by neutrophils.

miRNA	Effects/Interactions	Expression in OA	References
miR-9	Targets IL-6, NF-ΚB1 and MMP-13	Downregulated	[48,49]
miR-21	Inhibits GDF5	Upregulated	[50,51,52]
miR-29	Represses SOX9; targets collagen type 1A1	Upregulated	[50,51,52]
miR-30	Inhibits autophagy; induces apoptosis	Upregulated	[42,46]
miR-36	Induces expression of inflammatory factors and chemokines via NF-ΚB/A20 signaling	Upregulated	[53]
miR-92a	Inhibits histone deacetylase; involved in cartilage development and homeostasis	Downregulated	[50,54,55,56]
miR-95	Involved in cartilage development and homeostasis	Downregulated	[55]
miR-99a	Targets FZD1, ITGB5, GDF6; enhances inflammation and apoptosis	Downregulated	[47]
miR-100	Involved in cartilage homeostasis	Downregulated	[55]
miR-135b	Involved in chondrocyte proliferation and cartilage repair	Upregulated	[55,57]
miR-140	Induce SOX9, suppress hypertrophy by targeting SMAD1; involved in proliferation and migration of chondrocytes	Downregulated	[50,54,55]
miR-141	Inhibits bone resorption	Upregulated	[39]
miR-143	Targets genes including SMAD3 and DCAKD	Upregulated	[47]
miR-146a	Reduces Type II collagen; enhances autophagy and pro-inflammatory cytokines, inhibits NF-ΚB pathway; targets Camk2d	Upregulated	[42,50,58]
miR-155	Inhibits autophagy; overexpression reduces S100A8/19-P secretion of proinflammatory cytokines	Upregulated	[42,46,47]
miR-193a	Inhibit inflammation, apoptosis, and cartilage degradation mediators (MMP-3, MMP-13, and ADAMTS)-5; Targets SOX5	Downregulated	[55]
miR-204	Regulates osteogenesis by targeting Runx2 expression	Downregulated	[50,54]
miR-222	Regulates MMP-13 and histone deacetylase 4; improves chondrogenesis	Downregulated	[50,54]
miR-320c	Enhances chondrogenesis by upregulating SOX9 and downregulating MMP-13 and Wnt/β-catenin pathway	Downregulated	[55]
miR-370/373	Inhibits SHMT2 and MECP2	Downregulated	[50,54]
miR-381	Inhibits histone deacetylase	Downregulated	[50,54]
miR-490	Increases expression of Runx2, and decreases SOX9	Upregulated	[50,51,52]
miR-1271	Enhances expression of MMP-3, MMP-13 and ADAMTS-4, and decreases levels of SOX9, COL2A1 and aggrecan	Upregulated	[50,51,52]
